# Inclusion and definition of acute renal dysfunction in critically ill patients in randomized controlled trials: a systematic review

**DOI:** 10.1186/s13054-018-2009-x

**Published:** 2018-04-24

**Authors:** Rogerio da Hora Passos, Joao Gabriel Rosa Ramos, André Gobatto, Juliana Caldas, Etienne Macedo, Paulo Benigno Batista

**Affiliations:** 1 Critical Care Unit, Hospital São Rafael, Av São Rafael, Salvador, 2152 Brazil; 2 Department of Medicine, Division of Nephrology, University of California, San Diego, USA; 3 Critical Care Unit, Nephrology Department, Hospital Portugues, Salvador, Brazil

**Keywords:** Acute kidney injury, Critically ill, Intensive care unit, Mortality, Systematic review

## Abstract

**Background:**

In evidence-based medicine, multicenter, prospective, randomized controlled trials (RCTs) are the gold standard for evaluating treatment benefits and ensuring the effectiveness of interventions. Patient-centered outcomes, such as mortality, are most often the preferred evaluated outcomes. While there is currently agreement on how to classify renal dysfunction in critically ill patients , the application frequency of this new classification system in RCTs has not previously been evaluated. In this study, we aim to assess the definition of renal dysfunction in multicenter RCTs involving critically ill patients that included mortality as a primary endpoint.

**Methods:**

A comprehensive search was conducted for publications reporting multicenter randomized controlled trials (RCTs) involving adult patients in intensive care units (ICUs) that included mortality as a primary outcome. MEDLINE and PUBMED were queried for relevant articles in core clinical journals published between May 2004 and December 2017.

**Results:**

Of 418 articles reviewed, 46 multicenter RCTs with a primary endpoint related to mortality were included. Thirty-six (78.3%) of the trial reports provided information on renal function in the participants. Only seven articles (15.2%) included mean or median serum creatinine levels, mean creatinine clearance or estimated glomerular filtration rates. Sequential organ failure assessment (SOFA) score was the most commonly used definition of renal dysfunction (20 studies; 43.5%). Risk, Injury, Failure, Loss, End-stage renal disease (RIFLE), Acute Kidney Injury Network (AKIN) and Kidney Disease Improving Global Outcomes (KDIGO) criteria were used in five (10.9%) trials. In thirteen trials (28.3%), no renal dysfunction criteria were reported. Only one trial excluded patients with renal dysfunction, and it used urinary output or need for renal replacement therapy (RRT) as criteria for this diagnosis.

**Conclusion:**

The presence of renal dysfunction was included as a baseline patient characteristic in most RCTs. The RIFLE, AKIN and KDIGO classification systems were infrequently used; renal dysfunction was generally defined using the SOFA score.

## Background

Acute renal dysfunction affects one in five hospitalized patients [1] and occurs in up to 25% of critically ill individuals [2, 3]. Renal dysfunction is an independent risk factor for mortality, especially in patients treated with renal replacement therapy (RRT) [4]. Recent epidemiological studies have shown that renal dysfunction is associated with prolonged hospital stay, increased hospitalization costs, and progression to chronic kidney disease [2, 5].

Since 2004, the severity of kidney injury has been determined by several new classification systems: Risk, Injury, Failure, Loss, End-stage renal disease (RIFLE), Acute Kidney Injury (AKI) Network (AKIN) and Kidney Disease Improving Global Outcomes (KDIGO) [6]. These systems have provided a standardized assessment of renal dysfunction severity and consistent estimates of epidemiological measures [7, 8]. However, there is insufficient evidence to support their widespread application in critical care [9, 10]. Furthermore, in critically ill patients, renal dysfunction severity can also be evaluated by combining renal function with functional parameters of other organs (e.g., the Sequential Organ Failure Assessment (SOFA) score) [11].

In evidence-based medicine, multicenter, prospective, randomized controlled trials (RCTs) are the gold standard for evaluating treatment benefits and ensuring the effectiveness of interventions. Patient-centered outcomes, such as mortality, are most often the preferred evaluated outcomes [12]. While there is currently agreement on how to classify renal dysfunction in critically ill patients [13], the application frequency of this new classification system in RCTs has not previously been evaluated. In this study, we aim to assess the definition of renal dysfunction in multicenter RCTs involving critically ill patients that included mortality as a primary endpoint. In addition, we evaluated the criteria used to determine the severity and progression of kidney injury.

## Methods

### Search strategy and eligibility

A comprehensive search was conducted for publications reporting multicenter RCTs involving adult patients in intensive-care units (ICUs), with mortality as a primary outcome. The search was conducted in the MEDLINE database via the PubMed interface, including articles in the core clinical journals subset published May 2004 to December 2017 (In the list below). MEDLINE offers the “Core Clinical Journals” filter to limit searches to clinically useful journals [14, 15]. Eligibility assessment and data abstraction were performed independently in a non-blinded, standardized manner by two reviewers. Inter-rater reliability was evaluated using the kappa statistic. Discrepancies in methodological quality assessment and final classification of the RCTs were resolved by consensus among the authors. Comparison parameters included the definition and exclusion of patients with renal dysfunction, baseline serum creatinine levels, proportions of trial participants with renal dysfunction, and subgroup analyses involving acute renal dysfunction.

- 01 “intensive care”[MeSH Terms] OR Intensive care[Text Word]
- 02 “critical care”[MeSH Terms] OR critical care[Text Word]
- 03 (“critical illness”[TIAB] NOT Medline[SB]) OR “critical illness”[MeSH Terms] OR critically ill[Text Word]
- 04 “sepsis”[MeSH Terms] OR sepsis[Text Word]
- 05 “artificial respiration”[Text Word] OR “respiration, artificial”[MeSH Terms] OR mechanical ventilation[Text Word]
- 06 “adult respiratory distress syndrome”[Text Word] OR “respiratory distress syndrome, adult”[MeSH Terms] OR A RDS[Text Word]
- 07 (#01OR#02OR#03OR#04OR#05OR#06)
- 08 “randomized controlled trial”[Publication Type] OR “randomized controlled trials”[MeSH Terms] OR “randomized controlled trial”[Text Word] OR “randomised controlled trial”[Text Word]
- 09 #07 AND #08
- 10 (“Multicenter Studies”[MeSH] OR “Multicenter Study”[Publication Type]) OR multicenter[All Fields]
- 11. End Point Mortality
- 12 #09 AND #10

### Data extraction

The following data were extracted: (1) subject of study, (2) number of patients, (3) number of centers, (4) conditions studied, (5) allocation concealment, (6) exclusion of chronic kidney disease, (7) exclusion of acute renal dysfunction, (8) chronic kidney disease (CKD) criteria, (9) baseline acute renal dysfunction criteria, and (10) acute renal dysfunction as outcome (11) mortality. Two authors (RHP and PB) evaluated the selected studies for quality using the Consolidated Standards of Reporting Trials (CONSORT) checklist.

### Statistical analysis

Analyses were performed in SPSS 21.0 (SPSS, Inc.). Categorical variables are described as number (percentage).

## Results

**Fig. 1:**
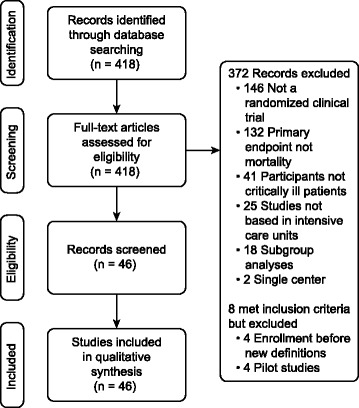
Flow diagram of studies assessed in the systematic review

The selection and exclusion of RCTs are summarized in Fig. 1. Inter-observer agreement among the reviewers for the selection and final classification of the studies was high, with a kappa statistic of 0.86. From 418 separate articles, 46 multicenter RCTs (including both single-continent and multi-continent settings) with a primary end point related to mortality were included [16–61]. Of these, 5 showed a beneficial effect of the trial intervention on mortality, whereas 41 demonstrated a neutral effect (Table 1).

**Table 1:** Description of the randomized controlled trials

Study	First author	Number	Centers	Area	Blinded	CKD excluded	Acute renaldysfunction excluded	CKD criteria	Baseline acute renal dysfunction criteria	Acute renal dysfunction as Outcome	Mortality
Norepinephrine plus dobutamine vs epinephrine in septic shock (2007)	D Annane [32]	330	19	Drug	Yes	No	No	McCabe	SOFA/RRT	NA	40.0%
Hydrocortisone in septic shock (2008)	CL Sprung [27]	499	52	Drug	Yes	No	No	NA	UO < 0.5 mL/kg/h for > 1 h or SOFA	NA	39.2%
Intensive insulin therapy in severe sepsis (2008)	FM Brunkhorst [30]	600	18	Drug	No	Yes	No	Yes	NA	2 × Cr or RRT	24.1%
PEEP setting in acute lung injury and ARDS (2008)	A Mercat [49]	767	37	MV	No	No	No	NA	SOFA	SOFA	31.2%
Vasopressin vs norepinephrine infusion in septic shock (2008)	JA Russell [37]	802	27	Drug	Yes	No	No	NA	SOFA	Brussels	43.9%
Ventilation strategy in acute lung injury and ARDS (2008)	MO Meade [52]	983	30	MV	Yes	No	No	NA	No	No	36.4%
Exogenous surfactant in acute lung injury and the ARDS (2009)	J Kesecioglu [26]	418	67	Drug	No	No	No	NA	SOFA or LODS	SOFA or LODS	24.5%
TAK-242 in severe sepsis (2010)	TW Rice [20]	274	93	Drug	Yes	Yes	No	NA	SOFA	SOFA	17.0%
Neuromuscular blockers in early ARDS (2010)	L Papazian [31]	340	20	Drug	Yes	No	No	NA	SOFA	SOFA or Cr >2	31.6%
Prone positioning in moderate and severe ARDS (2010)	P Taccone [50]	342	25	MV	No	No	No	NA	SOFA	SOFA	31.0%
Early lactate-guided therapy (2010)	TC Jansen [39]	344	4	Protocol	No	No	No	NA	SOFA	SOFA or RRT	33.9.0%
Corticosteroid and intensive insulin therapy in septic shock (2010)	D Annane [22]	509	11	Drug	No	No	No	NA	SOFA	SOFA	42.9.0%
Dopamine and norepinephrine in the treatment of shock (2010)	D De Backer [21]	1679	8	Drug	Yes	No	No	NA	SOFA	SOFA or RRT	52.0%
Recombinant tissue factor pathway inhibitor in severe community-acquired pneumonia (2011)	RG Wunderink [34]	2138	188	Drug	Yes	No	No	RRT	SOFA or Cr >3 or RRT	No	18.0%
Intravenous β-2 agonist in acute respiratory distress syndrome (2012)	SF Gao [53]	326	46	Drug	Yes	No	No	NA	No	Critical care minimum dataset	34.0%
IABP for myocardial infarction with cardiogenic shock (2012)	H Thiele [42]	600	37	Protocol	No	No	No	NA	UO <30 mL/h	GFR	39.7%
Hydroxyethyl vs Ringer’s acetate in severe sepsis (2012)	A Perner [28]	798	36	Drug	Yes	No	No	NA	SOFA	SOFA or RRT or 2 × Cr or RIFLE	51.0%
Drotrecogin alfa in septic shock (2012)	VM Ranieri [23]	1697	208	Drug	Yes	No	No	NA	SOFA	SOFA	26.4%
Hydroxyethyl vs saline for fluid resuscitation (2012)	JA Myburgh [29]	7000	32	Drug	Yes	Yes	Yes	NA	RIFLE	RIFLE	18.0%
Statin in patients with ventilator associated pneumonia (2013)	L Papazian [24]	300	26	Drug	Yes	No	No	McCabe	SOFA	No	21.2%
Recombinant human activated protein C in septic shock (2013)	D Annane [33]	411	24	Drug	Yes	No	No	McCabe	SOFA	SOFA	47.6%
Prone positioning in ARDS (2013)	C Guérin [51]	474	27	MV	No	No	No	McCabe	SOFA	SOFA	32.8%
High-frequency oscillation for early ARDS (2013)	ND Ferguson [48]	500	27	MV	No	No	No	NA	No	No	47%
High-frequency oscillation for ARDS (2013)	D Young [47]	795	29	MV	No	No	No	NA	No	No	41.7%
Effect of early vs late tracheostomy (2013)	D Young [41]	909	72	Protocol	No	No	No	NA	No	No	30.8%
Glutamine and antioxidants in critically ill patients (2013)	D Heyland [38]	1223	40	Nutrition	Yes	No	No	NA	Cr >2 or UO <500 mL/24 h or > 80 baseline	SOFA	32.4%
Early parenteral nutrition in critically ill patients (2013)	GS Doig [16]	1372	31	Nutrition	No	No	No	RRT	No	SOFA or RRT	22.8%
Colloids vs crystalloids in hypovolemic shock (2013)	D Annane [25]	2857	57	Drug	No	Yes	No	McCabe	SOFA	SOFA	25.4%
Perioperative goal-directed hemodynamic optimization in abdominal surgery (2014)	D Pestaña [43]	132	6	Protocol	No	No	No	NA	No	No	4.2%
Perioperative, cardiac output-guided hemodynamic therapy algorithm in gastrointestinal surgery (2014)	RM Pearse [40]	734	17	Protocol	Yes	No	No	Cr >1.4	No	2× Cr or <0.5 mL/12 h	33.6%
Rosuvastatin for sepsis-associated ARDS (2014)	NHLBI ARDS Network [35]	745	44	Drug	Yes	No	No	RRT	SOFA	SOFA	28.5%
High vs low blood-pressure target in septic shock (2014)	P Asfar [36]	776	29	Drug	No	No	No	RRT	Cr > 1.9 or 500 ml/24 h	2c Cr or RRT	34.0%%
Lower vs higher hemoglobin in septic shock (2014)	LB Holst [45]	998	32	Transfusion	No	No	No	RRT	SOFA	NA	43.0%%
Album replacement in patients with severe sepsis or septic shock (2014)	P Caironi [19]	1781	100	Drug	No	No	No	NA	SOFA	SOFA	43.6%
Trial route of early nutritional support in critically ill adults (2014)	SE Harvey [18]	2388	33	Nutrition	No	No	No	RRT	SOFA	RRT	33.1%
Noninvasive ventilation vs oxygen therapy in immunocompromised patients with ARDS (2015)	V Lemiale [46]	374	28	MV	No	No	No	Charlson	SOFA	SOFA	24.1%
Permissive underfeeding or standard enteral feeding (2015)	YM Arabi [17]	894	7	Nutrition	No	No	No	NA	SOFA	SOFA	27.2%
Age of transfused blood in critically ill adults (2015)	J Lacroix [44]	2510	64	Transfusion	Yes	No	No	NA	MODS	MODS	37%
Renal-replacement therapy in the intensive care unit (2016)	S Gaudry [54]	620	31	RRT	No	No	Yes	No	KDIGO	KDIGO/RRT	48.5%
Sodium selenite and procalcitonin-guided therapy in severe sepsis or septic shock (2016)	F Bloos [55]	1180	33	Drug	No	No	No	No	No	No	28.0%
Quality improvement intervention with daily round in critically ill patients (2016)	AB Cavalcanti [56]	6877	118	Daily check list	No	No	No	No	No	No	32.9%
Levosimendan after cardiac surgery (2017)	G Landoni [58]	548	14	Drug	Yes	Yes	No	No	No	RIFLE	12.9%
Recruitment and titrated PEEP vs low PEEP on mortality in ARDS (2017)	ART Group [57]	1008	9	MV	No	No	Yes	NA	No	No	55.0%
Age of red cells for transfusion and outcomes in critically ill (2017)	DJ Cooper [59]	4919	5	Transfusion	Yes	Yes	No	RRT	KDIGO	KDIGO	24.8%
Restrictive vs liberal transfusion in cardiac surgery (2017)	CD Mazer [60]	5243	73	Transfusion	No	Yes	No	Omitted	KDIGO	KDIGO	30.0%
Short-term vs long-term blood storage on mortality (2017)	NM Heddle [61]	24,743	4	Transfusion	No	No	No	No	SOFA	No	9.1%

*CKD* chronic kidney disease, *PEEP* positive end-expiratory pressure; *SOFA* sequential organ failure assessment, *MV* mechanical ventilation, *MODS* multiple organ dysfunction score, *GFR* glomerular filtration rate; *LODS* logistic organ dysfunction score, *UO* urine output, *RRT* renal replacement therapy, *KDIGO* Kidney Disease Improving Global Outcomes; *RIFLE* Risk, Injury, Failure, Loss of kidney function, End-stage kidney disease, *TAK-242* a small-molecule inhibitor of Toll-like receptor-4-mediated, *ARDS* acute respiratory distress syndrome, *IABP* intraaortic balloon pump, *Cr* creatinine

**Fig. 2:**
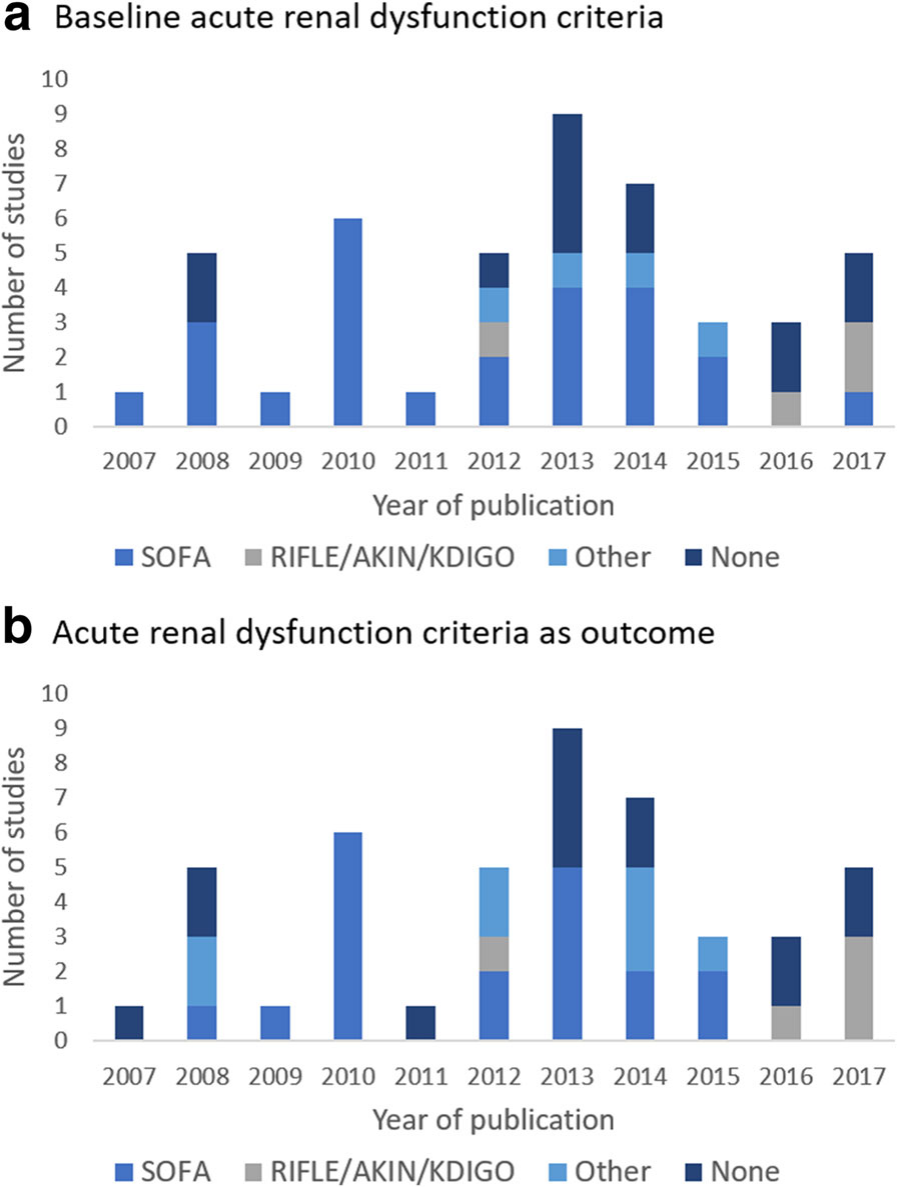
Number of studies per year of publication stratified by baseline acute renal dysfunction criteria (**a**) and acute renal dysfunction criteria as outcome (**b**). SOFA, Sequential Organ Failure Assessment; RIFLE, Risk, Injury, Failure, Loss, End-stage renal disease; AKIN Acute Kidney Injury Network; KDIGO, Kidney Disease Improving Global Outcomes

The distribution of the number of studies per year of publication, stratified by the acute renal dysfunction criteria used as a baseline and outcome measure is described in Figs. 2a and b, respectively (Fig. 2).

### Conditions studied

A wide range of conditions in critically ill patients was studied in the 46 RCTs, including sepsis (13 RCTs), acute respiratory distress syndrome (11 RCTs), shock (5 RCTs), nutrition (4 RCTs), anemia (5 RCTs), surgery (3 RCTs), respiratory failure (2 RCTs), pneumonia (2 RCTs), renal replacement therapy (1 RCT), and quality improvement (1 RCT).

### Interventions

The RCTs assessed a range of interventions in critically ill patients (Table 1), including drug treatment (22 RCTs), nutrition (4 RCTs), hemodynamic optimization (5 RCTs), transfusion (5 RCTs), mechanical ventilation (8 RCTs), timing of renal replacement therapy (1 RCT) and daily round checklist (1 RCT).

### Reporting of acute renal dysfunction in cohort characteristics

Thirty-six trial reports (78.3%) provided information on acute renal (dys)function in the participants. Only seven articles (15.2%) contained mean or median serum creatinine levels, mean creatinine clearance or estimated glomerular filtration rates (eGFRs). The SOFA score was the most commonly used definition of acute renal dysfunction, in 20 studies (43.5%): RIFLE/AKIN/KDIGO criteria were used in 5 trials (10.9%). In thirteen trials (28.3%) no criteria for defining acute renal dysfunction were reported. Only one trial (2.2%) excluded patients with acute renal dysfunction, using urinary output or need for RRT as criteria for this diagnosis. As shown in Fig. 2, RIFLE/KDIGO/AKIN criteria were mostly used in recent years (2016 and 2017).

### Reporting of acute renal dysfunction in secondary outcomes

Most of the trials studied acute renal dysfunction as a secondary outcome, which was reported in 33 trials (71.7%). The renal SOFA score was the most commonly used definition, in 19 trials (41.3%), followed by the need for RRT, used in 10 trials (21.7%) and RIFLE/AKIN/KDIGO criteria used in 5 trials (10.9%). Only six articles (13.0%) included serum creatinine levels, mean creatinine clearance, or GFR (eGFR) values as secondary outcomes.

Five trials (10%) reported progression to more severe stages of acute renal dysfunction. No trial reported progression to chronic kidney disease. Thirty-three trials (71.7%) evaluated organ dysfunction in addition to renal dysfunction.

## Discussion

Our results demonstrated that patients with acute renal dysfunction were often included in multicenter RCTs involving critically ill patients that included mortality as a primary endpoint. However, current classification systems, such as RIFLE/AKIN/KDIGO, were not frequently used to define renal dysfunction in the descriptions of patient baseline characteristics or as secondary outcomes.

Despite the advances from widespread use of new classification systems and the development of new biomarkers for early renal dysfunction detection, little progress has been made in developing evidence-based interventions for renal dysfunction prevention and treatment [10]. For critically ill patients, the lack of positive results may be related to the parameters used to measure renal function, primarily creatinine concentration and urine output, because these parameters are frequently influenced by comorbidities, nutritional status, fluid overload and the overall severity of critical illness [62].

A single definition of acute renal dysfunction would be useful for clinical practice, research, and public health [13]. This definition has been rapidly changing in the literature since 2004 with the introduction of the RIFLE, AKIN, and KDIGO classification systems. These classifications were developed based on both evidence and consensus [63]. However, our findings show that with a few exceptions, they were not applied in RCTs with mortality as a primary outcome published in the period of this study. Furthermore, these systems were not widely used for defining or evaluating renal dysfunction as a secondary endpoint. These findings may raise concerns about the evidence-based use of these classification systems in the clinical management of critically ill patients. Nevertheless, it is important to notice that there was an apparent increase in the utilization of these scores in recent years (2016 and 2017).

Although the acute renal dysfunction (RIFLE/AKIN/KDIGO) classification systems have been compared and validated [64], they do have certain limitations. First, the use of small changes in serum creatinine levels to diagnose AKI is limited by the high rates of false-positive diagnoses caused by the inherent variability of serum creatinine levels in patients with higher baseline values, thus potentially misclassifying patients with CKD [65]. Second, in contrast to individual measurements, efforts to determine the trajectory of serum creatinine levels can identify AKI sub-phenotypes with different mortality risks, even among patients with AKI of similar severity. These AKI sub-phenotypes might define patients at risk of poor outcomes (i.e., those with non-resolving AKI), who might benefit from novel interventions [66]. Third, renal dysfunction definitions that require a reference creatinine value to analyze baseline renal function should utilize a value that reflects steady-state kidney function prior to an AKI episode. When such reference values are not available, surrogate estimates are required, and these can affect the accuracy of the determination [67]. In contrast, the simplicity of the SOFA score and the objectivity of the variables required for its calculation make it useful for repeated measurements of the degree of organ dysfunction or failure [68].

The renal SOFA score was the most commonly used system to quantify renal function at baseline or as a secondary outcome. It may be more convenient to study changes in the SOFA score over time. Such changes have been assessed in critically ill patients over 48 h [69] or during treatment [70] and have also been used to evaluate the degree of organ dysfunction in sepsis [63]. In addition to assessing patient status, renal criteria can be used for prognosis. An early and sequential evaluation pattern (using any of the various scoring systems) has been shown to be a superior approach for prognostic scoring in critically ill patients who develop renal dysfunction compared with a single assessment at any time point during an ICU admission or stay [71]. Similarly, in patients with kidney injury, measuring changes in the SOFA score in the first 24 h of RRT can identify patients at high risk of mortality [72]. In contrast, individual SOFA scores are poor at predicting early (7 day) mortality in patients with septic AKI who require continuous RRT [73].

In addition to the new definitions of renal dysfunction, the SOFA score has been validated as a tool for assessing sequential organ dysfunction and is a good prognostic indicator. Furthermore, this score is familiar to critical care physicians and has been used for years in critical care settings and for different clinical conditions [69]. To date, no study has directly compared SOFA with RIFLE/AKIN/KDIGO; however, the use of RIFLE criteria improved the performance of the Acute Physiology and Chronic Health Evaluation disease classification system II (APACHE II) score in predicting mortality in critically ill patients [74]. The prognostic value of a hypothetical score that combines RIFLE/AKIN/KDIGO criteria with the SOFA score, perhaps by replacing renal SOFA criteria variables with KDIGO criteria variables, is a matter of future research.

To our knowledge, the present manuscript is the first to describe the characterization of acute renal dysfunction in RCTs of critically ill patients. We have utilized an extensive search covering a period of 13 years following publication of current renal dysfunction definitions.

Nonetheless, our study does have several limitations. First, we have limited our sample to papers published in high-impact journals because these are typically multicenter studies with a better opportunity for impacting clinical practice [75]. We defined high-impact journals as those included in the MEDLINE core clinical journals subset [14]. The core clinical journals subset is an easy filter to apply. Using this filter, a MEDLINE search can focus on a set of journals selected for high quality and clinical utility, which may aid in the reproducibility of our findings, though we do recognize that there have been controversies as to the actual clinical utility of this subset [15]. However, because the new definitions of renal dysfunction are used at similar frequencies among the major journals, it is reasonable to assume that the addition of extra journals would not have meaningfully changed our results. In agreement with our methodology, other recently published reviews have utilized similar procedures [76, 77]. Nevertheless, this selection procedure may result in biases because papers published in journals with a lower impact factor may characterize renal dysfunction differently. Another limitation is that because we relied on published material as the typical information source for clinicians, we cannot exclude the possibility that some trials reported characterizations of renal dysfunction that differed from their original protocols [78].

## Conclusion

The presence of renal dysfunction was included as a baseline patient characteristic and as an outcome measure in most multicenter RCTs involving critically ill patients with mortality as a primary endpoint that were published in core clinical journals in the study period. The analyzed RCTs generally defined acute renal dysfunction using the SOFA score, with a less frequent utilization of the RIFLE, AKIN and KDIGO classification systems. There is a need for further evaluation of the validity and barriers for utilization of each score to better inform clinical practice.

## Abbreviations

AKI: Acute kidney injury
AKIN: Acute Kidney Injury Network
APACHE: Acute Physiology and Chronic Health Evaluation disease classification system
CKD: Chronic kidney disease
eGFR: Estimated glomerular filtration rate
GFR: Glomerular filtration rate
ICUs: Intensive care units
KDIGO: Kidney Disease Improving Global Outcomes
RCTs: Randomized controlled trials
RIFLE: Risk, Injury, Failure, Loss, End-stage renal disease
RRT: Renal replacement therapy
SOFA: Sequential Organ Failure Assessment
